# Case Report: Kawasaki Shock Syndrome With Polycyclic Eruption: A Peculiar Brain Imaging

**DOI:** 10.3389/fped.2021.651457

**Published:** 2021-10-15

**Authors:** Enrico Masiello, Danilo Buonsenso, Ilaria Lazzareschi, Antonio Gatto, Marco Piastra, Antonio Chiaretti, Piero Valentini

**Affiliations:** ^1^Pediatric Unit Dono Svizzero Hospital, Formia, Italy; ^2^Department of Woman and Child Health and Public Health, Fondazione Policlinico Universitario A. Gemelli IRCCS, Rome, Italy; ^3^Department of Pediatric ICU, Intensive Care and Anesthesia, Fondazione Policlinico Universitario A. Gemelli IRCCS, Rome, Italy

**Keywords:** Kawasaki disease, Kawasaki disease shock syndrome, CNS inflammation in Kawasaki disease shock syndrome, CNS involvement in Kawasaki disease, MISC

## Abstract

Kawasaki disease (KD) is a childhood vasculitis of unknown etiology. The present study describes a case of KD shock syndrome that occurred in an infant (age, 16 months) following 7 days of high fever and persistent rash characterized by target-like and purpuric skin lesions. The child developed neurological manifestations such as altered consciousness and irritability. Consequently, brain magnetic resonance imaging (MRI) was performed, revealing an inflammatory involvement of the anterior perforated substance and the hypothalamus. Cerebral involvement on brain MRI is rarely described in KD but when reported is characterized mostly by cerebral vasculitis. We illustrate for the first time in KD an inflammation in the brain not related to vasculitis, reporting peculiar neuroradiological findings. This last aspect has fascinated us in light of recent evidence about the immunological spectrum of Multisystem Inflammatory Syndrome in Children (MIS-C) and Kawasaki-like syndrome in the Severe Acute Respiratory Syndrome Coronavirus 2 (SARS-CoV-2) outbreak.

## Introduction

**Figure 1:**
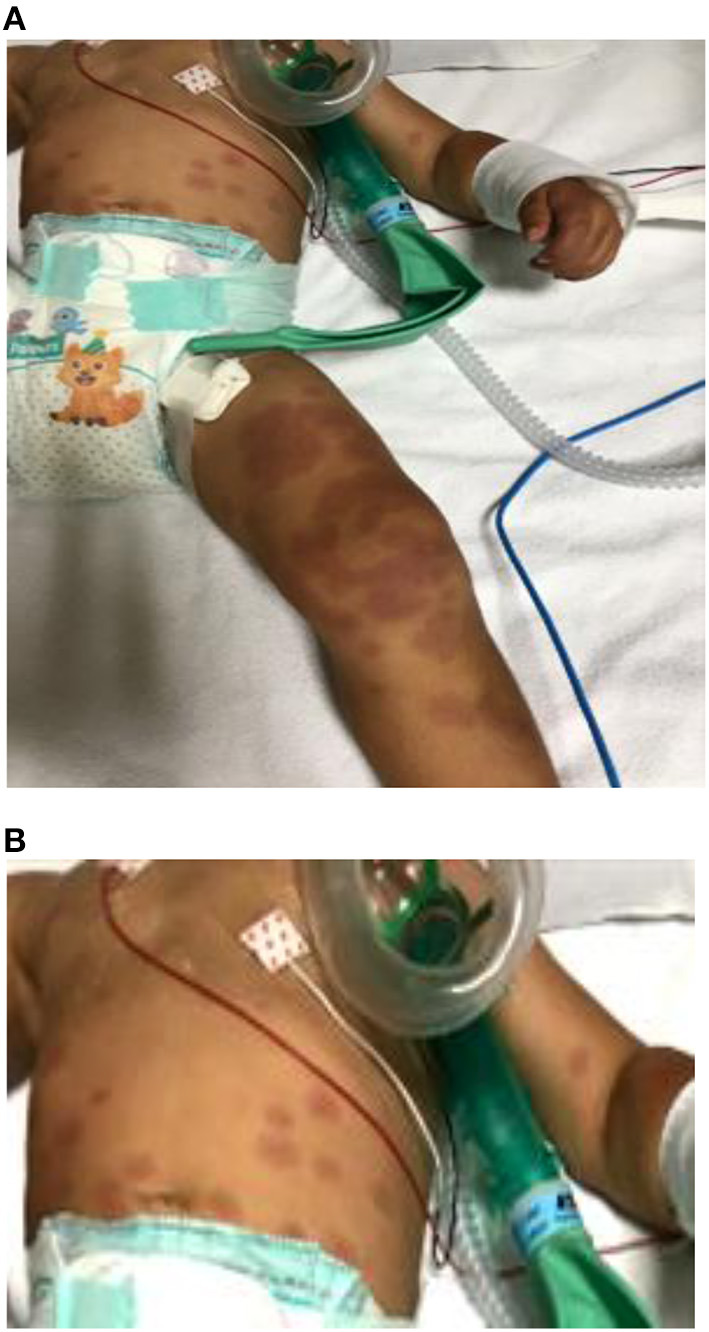
**(A)** Erythematous round-shaped (polycyclic appearance) rash on the legs. **(B)** Target-like rash on the abdomen with the center of the lesion darker then the periphery. Similar lesions were present on the face and arms.

A previously healthy 16-month-old male child was transferred to our hospital for continuous high-grade fever associated with erythematous and target-like rash (Figure 1) 5 days after the beginning of fever.

At the local hospital, on clinical examination neurological symptoms such as irritability and lethargy were noted; blood examinations showed leukocytosis with neutrophilia (white blood cell count: 21,000/μL) and elevated procalcitonin (PCT: 15.6 ng/mL; n.v: normal values < 0.5) and C-reactive protein (CRP: 8.9 mg/L; n.v: normal values < 5) levels. Intravenous (IV) fluids and ceftriaxone had been immediately administered for suspected meningococcemia.

On admission to our unit, a body temperature of 39.6°C was recorded. The boy appeared extremely irritable, and physical examination demonstrated an erythematous eruption with round lesions characterized by polycyclic contours covering the limbs and the abdomen with many targeted lesions involving the abdomen, the arms, and the face. Bilateral erythema and edema of palms and soles were also described (Figure 1). His mother reported the beginning of the rash 2 days after the onset of fever. Initially, erythematous-purpuric lesions on the limbs were noted, spreading confluently later to the inferior abdomen, where some peripheral targeted lesions were also present. Similar well-delineated, target-like lesions appeared in the upper abdomen quadrants and subsequently on his face and arms.

Cervical lymph nodes were not swollen on clinical evaluation. The physical examination showed a strawberry tongue and intense pharyngeal hyperemia, although the conjunctiva and the lips appeared normal.

The peripheral blood test exhibited leukocytosis (white blood cell count, 11,660 cells/μL, with 82.6% neutrophils), thrombocytosis (platelets 777,000 cells/μL), and increased fibrinogen (772 mg/dL, n.v: normal values < 500) and d-dimer (13,030 ng/mL, n.v: normal values 200–400) with partial thromboplastin time and activated partial thromboplastin time values in the normal range. Marked elevated levels of CRP (98 mg/L; n.v: normal values < 0.5) and PCT (54 ng/mL; n.v: normal values < 0.5) were seen.

Lumbar puncture was performed and cerebrospinal fluid (CSF) analysis was normal. The antibiotic therapy (ceftriaxone) was continued, and acyclovir was prescribed until results of microbiological analyses in the CSF were available.

Reverse transcriptase–polymerase chain reaction (RT-PCR) analyses for detection of cytomegalovirus (CMV), Epstein–Barr virus (EBV), polyomavirus JC, enterovirus, herpes simplex virus, human herpesvirus type 6 (HHV-6), and varicella zoster virus genome in CSF came back negative. RT-PCR analyses for detection of the enterovirus genome in stool and pharyngeal swabs gave negative results. Suspecting a viral infection, serological tests for adenovirus, EBV, CMV, coxsackievirus, influenza A and B, human parainfluenza (1–3) virus, and parvovirus B19 were performed and came back negative too. The urinary examination revealed sterile pyuria and mild proteinuria. CSF, blood, and pharyngeal swab cultures were also negative.

On the second day of hospitalization, we documented sudden-onset drowsiness with progressive lethargy. Brain computed tomography (CT) was performed, giving a negative result. The infant developed tachycardia, systolic hypotension (60/50 mm Hg), oliguria, and mild dyspnea, with cold extremities a few hours later.

An initial echocardiographic examination revealed first-degree mitral valve regurgitation and left ventricular hypokinesia with pericardial effusion (3 mm in thickness). Hyperechogenic coronary arteries with a normal size were also documented. Ejection fraction (EF) was estimated to be 35–40%. A chest X-ray revealed a small retrocardiac consolidation.

The development of hypotension, despite massive IV fluids resuscitation, associated with the neurological deterioration of the child required inotropic support (dobutamine infusion) given until the second dose of IV immunoglobulin (IVIG) was administered and continuous monitoring in the intensive care unit.

On the second day, suspecting KD shock syndrome (KDSS), a first dose of IVIG was administered (2 g/kg) but not effective on the fever spikes. On the same day, oral aspirin was given in an anti-inflammatory dose (100 mg/kg per day for 3 days then at a dose of 30 mg/kg per day).

Abdominal ultrasound demonstrated a hydropic gallbladder and mild ascitic effusion surrounding the liver and in the pelvis. On the fourth day, a second dose of IVIG was prescribed with clinical improvement and effect on the fever spikes.

Hypotension regressed after 1 day of dobutamine infusion. EF increased after the first dose of immunoglobulins and further after the second dose.

Interestingly, the rash was initially mitigated by the first dose of IVIG, but the second was more effective with simultaneous blanching in the areas concerned. Echocardiographic examination on the 6th day revealed exclusively residual and persistent hyperechogenicity of the coronary arteries.

Blood tests showed a decreased hemoglobin level (from 11 to 7.6 g/dL) requiring blood transfusion.

Serum immunoglobulin levels were normal. Albumin (ALB) was decreased (total protein: 61 g/L, reference range: 57–80 g/L; ALB: 27 g/L, reference range: 37–51 g/L); total bilirubin (TBIL) (TBIL: 0.2 mg/dL; reference range: 0.3–1.2 mg/dL), alanine aminotransferase, and aspartate aminotransferase were normal. Other blood biochemical tests, including glucose, triglycerides, and calcium were all within reference limits. Renal function and electrolytes were normal; the myocardial enzyme spectrum was also normal, but an increased level of N-terminal prohormone brain natriuretic peptide was evidenced (26,696 pg/mL, n.v: normal values < 150), confirming an ongoing cardiac dysfunction.

The throat culture, anti–streptolysin O, and anti–DNase B came back negative; therefore, the hypothesis of streptococcal infection was discarded.

**Figure 2:**
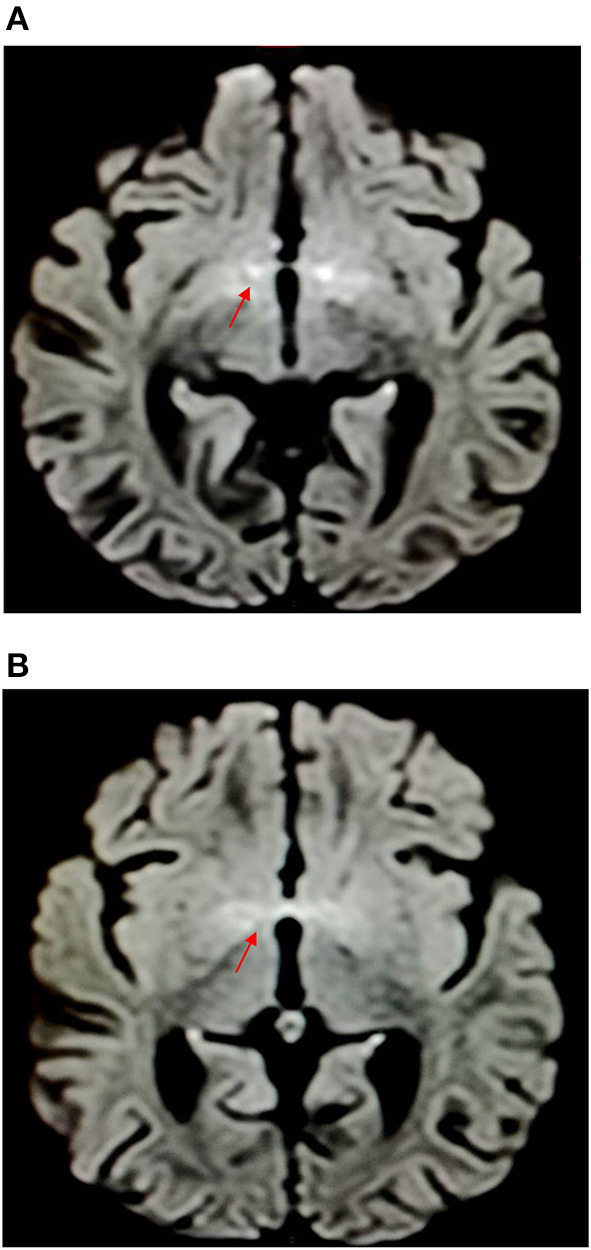
Hyperintensities in diffusion-weighted imaging at the level of the anterior perforated substance and hypothalamus.

**Figure 3:**
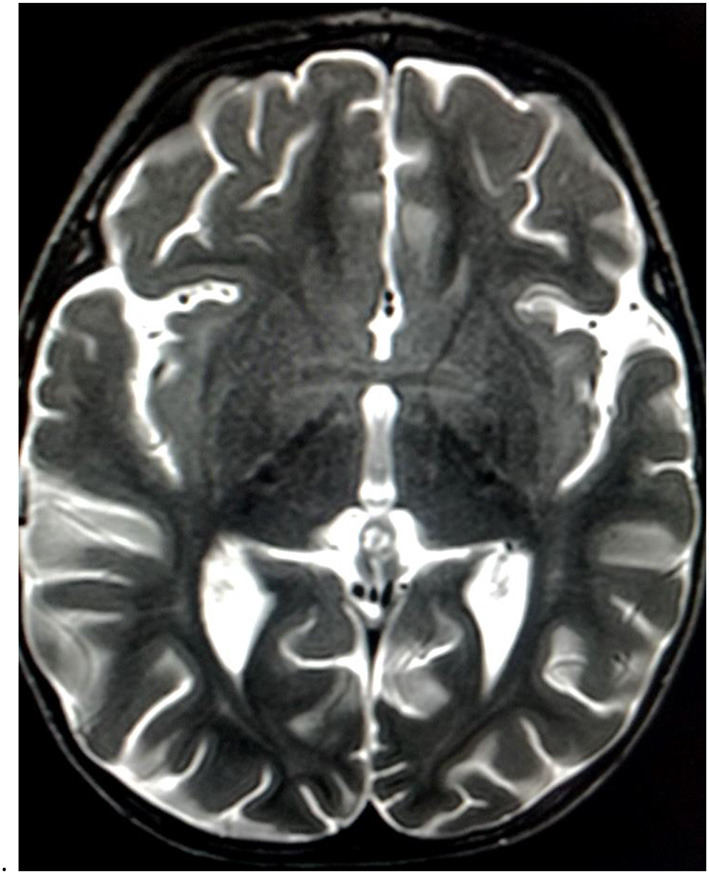
Mild T2 hyperintensities at the level of the anterior perforated substance and hypothalamus.

Brain magnetic resonance imaging (MRI) was performed 1 week later after the admission to rule out a neurological disease, showing hyperintensities in diffusion and T2-weighted imaging at the level of the anterior perforated substance and hypothalamus, with proton density reduction only in the anterior commissure (Figures 2, 3). Radiologists had suggested an underlying inflammatory/postinfectious process (related to HHV-6 virus), a hypothesis not supported by diagnostic tests (Figures 2, 3). A retropharyngeal abscess (diameter: 10 × 15 mm) with phlogistic involvement of the next longus colli muscle was also described on brain MRI scans, likewise previously reported in KD in literature.

To rule out a vasculitic rash complement levels, antineutrophil cytoplasmic antibody, antinuclear antibody, and anticardiolipin antibody tests were studied and came back normal, such as C3 and C4 levels.

Brain MRI without lesions in the white matter and multifocal involvement, CSF negative results without pleocytosis, and absent history of past infection/vaccination and/or demyelinating disorders led us to exclude acute disseminated encephalomyelitis. The patient has been followed for 12 months without any neurological and/or cardiac complications as confirmed by subsequent brain and cardiac MRI. Pituitary disorders were excluded.

## Discussion

Kawasaki disease (KD) is a vasculitis predominantly affecting those in infancy and early childhood. The etiology remains unknown. An abnormal and exaggerated inflammatory response to infectious triggers in genetically susceptible individuals is widely considered as the major cause of KD (1).

Coronary artery damage represents the main complication, and the therapy is intended to prevent coronary artery aneurysms (2). In this case report, KDSS was recognized when a change in mental status associated with hypotension occurred, prompting Doppler echocardiography.

According to current guidelines, we made a diagnosis of atypical KD based on laboratory evaluation and cardiac imaging (criteria for typical KD were not all satisfied: fever lasting at least 5 days and the presence of four of five main clinical features such as polymorphous rash, bilateral non-exudative conjunctivitis, changes in the extremities, changes of lips and oral cavity, and cervical non-purulent lymphadenopathy) (3).

By contrast, we reported fever lasting more than 7 days, associated with rash, bilateral erythema, and edema in the palms and soles, in addition to very high CRP levels. Also, other typical laboratory findings (anemia, thrombocytosis, hypoalbuminemia, and sterile pyuria) were positive in this child, and hydrops of the gallbladder and retropharyngeal abscess was described.

KDSS is a rare manifestation of KD (3, 4).

Kanegaye et al., in 2009, defined KDSS as the presence of any of the following conditions: a sustained decrease in systolic blood pressure (≥20% compared to normal range) or clinical signs of poor perfusion (4).

In a cohort of 187 children with KD, the prevalence of KDSS was 7%, with a higher prevalence of coronary artery abnormalities (62 vs. 23%) and IVIG resistance (46 vs. 18%) in KDSS than KD without shock (4). Our patient required two doses of IVIG.

KDSS may be initially mistaken for septic shock; therefore, this condition must be recognized, particularly in children younger than 6 months (incomplete KD is more frequent in early infancy), in order to perform an echocardiogram and begin appropriate treatment (4).

Transthoracic echocardiography in our case indeed showed ventricular hypokinesia, pericardial effusion, and reduced EF, findings only occasionally seen in KD. The development of KDSS should be suspected, particularly when left ventricular dysfunction is present (4).

Interestingly, the cardiovascular disturbance recovered promptly after specific therapy.

Involvement of the central nervous system (CNS) is a peculiar aspect of this report. Extreme irritability is described in KD but initially, in this child, was misinterpreted as meningoencephalitis. Neurological manifestations such as lethargy are rarely described in KD. Children experience this complication early (a transitory state of lethargy was documented at the local hospital) and generally, such as in this case, with an excellent prognosis (5).

In a recent retrospective study, neurological involvement was reported most frequently in KDSS than in KD (*p* < 0.001). Headache, irritability, somnolence, convulsions, signs of meningeal irritation, and facial nerve palsy in KD may warrant a complete diagnostic evaluation to exclude KDSS. Moreover, these symptoms can occur in the first phase of the disease and merit accurate evaluation in children affected by KD to make a diagnosis of KDSS early (6).

Brain MRI revealed hyperintensities in the hypothalamus and anterior perforated substance, findings never reported before (for characteristics and location) and probably related to CNS inflammation (Figure 1) despite the fact that CT and CSF chemical tests were normal. It is unclear if neurological symptoms could be more frequent in KDSS due to a “cytokine storm,” more intense in KDSS than in KD without shock (7).

To the best of our knowledge, this is the first time these radiological features on brain MRI have been reported in literature and may represent a new concept of cerebral involvement in KD. Previous studies described KD as a matter injury determined by vasculitic lesions with microhemorrhages (8–11).

In this case report, the radiologist, contrary to previous literature, instead of vascular hypoperfusion damage described an inflammatory disorder of the brain (particularly in diencephalic regions).

In the recent outbreak of the 2019 novel coronavirus, similar cases characterized by multiorgan failure and Kawasaki-like disease, both with relevant CNS involvement, as a part of a “postviral” systemic inflammatory disease, were described, in the context of the Multisystem Inflammatory Syndrome in Children (MIS-C) spectrum.

KDSS shares many features with MIS-C such as the risk of multiorgan failure, although in the first case it could be explained almost completely in light of typical myocardial dysfunction, whereas the same aspect in MIS-C could be more complex with systemic damage directly caused by an exaggerated adaptive immunity against coronavirus disease 2019 (COVID-19) and the following coagulation disorder. A powerful inflammatory response after infection by severe acute respiratory syndrome coronavirus 2 (SARS-CoV-2) is thought to be crucial (12). Also, in our case, which occurred in 2018, MRI demonstrated an “inflammatory storm” in the brain and peculiar imaging.

At present, more than in the past, the description of neurological symptoms and a generalized cardiac involvement in children affected by Kawasaki like–disease warrant the need for a careful clinical management and exclusion of COVID-19 infection. The presence of encephalopathy, similarly as in this case, in children affected by SARS-CoV-2, is thought to be predictive of multiorgan involvement and Kawasaki-like course (12, 13).

It must be underlined that in our case encephalopathy was present at the local hospital, some days before the onset of hypotension. Moreover, skin lesions similar to erythema multiform were reported in children with SARS-CoV-2 infection (14).

We have not performed COVID-19 serology despite clinical presentation because in our opinion it would be not determinant, considering that this episode was reported more than 1 year before the beginning of the SARS-CoV-2 pandemic and the uncertain long-term persistence of SARS-CoV-2 immunoglobulin G response. Conversely, we think that similar cases in the past might be reevaluated in light of the new knowledge acquired in this pandemic.

The importance of a better clinical and immune-pathology characterization in these episodes is even clearer. Moreover, we described peculiar neuroradiological findings whose significance needs to be better understood. The limitation of this study is that only one patient has been studied. Further clinical trials including a detailed assessment of encephalopathy and an accurate characterization of dermatological lesions, in the even more challenging spectrum of KD, are needed in order to recognize pathological pathways, different levels of inflammatory activation, and hopefully specific targets for common therapeutic strategies in different diseases.

## Data Availability Statement

The raw data supporting the conclusions of this article will be made available by the authors, without undue reservation.

## Ethics Statement

Written informed consent was obtained from the minor(s)' legal guardian/next of kin, for the publication of any potentially identifiable images or data included in this article.

## Author Contributions

EM and DB: conceived, designed, and wrote the report. IL, AG, MP, and AC: collected clinical data about the case. EM, DB, and PV: collected literature related to the case, conceived analogies between MISC and KDSS, and identifyed the importance of brain MRI results. All authors contributed to the article and approved the submitted version.

## Conflict of Interest

The authors declare that the research was conducted in the absence of any commercial or financial relationships that could be construed as a potential conflict of interest.

## Publisher's Note

All claims expressed in this article are solely those of the authors and do not necessarily represent those of their affiliated organizations, or those of the publisher, the editors and the reviewers. Any product that may be evaluated in this article, or claim that may be made by its manufacturer, is not guaranteed or endorsed by the publisher.
